# Unicompartmental Knee Arthroplasty in Spontaneous Osteonecrosis of the Knee: A Narrative Review of Indications, Techniques, and Outcomes

**DOI:** 10.7759/cureus.93859

**Published:** 2025-10-05

**Authors:** Zubair Younis, Muhammad A Hamid, Thomas Devasia, Faliq Abdullah

**Affiliations:** 1 Orthopaedics, The Royal Wolverhampton NHS Trust, Wolverhampton, GBR; 2 Orthopaedic Surgery, University Hospitals Birmingham NHS Foundation Trust, Birmingham, GBR; 3 Trauma and Orthopaedics, The Royal Wolverhampton NHS Trust, Wolverhampton, GBR; 4 Trauma and Orthopaedics, University Hospital of Wales, Cardiff, GBR

**Keywords:** outcome, partial knee replacement, spontaneous osteonecrosis, spontaneous osteonecrosis of the knee (sonk), unicompartmental knee arthroplasty

## Abstract

Spontaneous osteonecrosis of the knee (SONK) is a painful condition characterized by localized subchondral bone necrosis, predominantly affecting the medial femoral condyle in older adults. While total knee arthroplasty (TKA) is a definitive solution, unicompartmental knee arthroplasty (UKA) has emerged as a bone and ligament preserving alternative for this typically unicompartmental disease. This narrative review synthesizes current evidence on the indications, techniques, and outcomes of UKA for SONK. In well-selected patients, those with isolated condylar involvement, intact ligaments, correctable deformity, and a lesion size <50% of the condyle, UKA demonstrates excellent outcomes, including significant improvements in knee scores, high patient satisfaction (91-97%), and favorable 10-year survivorship (89-97%). Advantages over TKA include faster recovery, lower morbidity, and preservation of native kinematics. However, success is contingent upon strict adherence to these selection criteria, as UKA is contraindicated in inflammatory arthritis, metaphyseal extension, or significant patellofemoral arthritis. Technical execution must address challenges like bone defects and precise component alignment to mitigate risks of bearing dislocation or subsidence. Ultimately, UKA represents a successful joint-preserving strategy for appropriate candidates with SONK, although future work is needed to establish long-term data and refined guidelines.

## Introduction and background

Spontaneous osteonecrosis of the knee (SONK), first described by Ahlbäck et al. in 1968, is a distinct clinical entity characterized by sudden-onset knee pain, often worse at night or during weight-bearing, predominantly affecting adults over 50 years of age [1]. It typically involves localized subchondral bone necrosis, most commonly in the medial femoral condyle (MFC), with limited periarticular involvement and minimal degenerative changes in other compartments [1]. The etiology remains incompletely understood, although associations with varus malalignment, meniscal pathology, and subchondral insufficiency fractures have been proposed [2]. SONK exhibits a female predominance (3:1 ratio) and progresses through distinct radiological stages, with advanced stages often featuring condylar collapse and secondary osteoarthritis [1]. 

Historically, treatment paradigms evolved from conservative management (for early stages) to joint-preserving procedures (e.g., core decompression, high tibial osteotomy (HTO), or arthroplasty) [3]. Total knee arthroplasty (TKA) became a standard for advanced disease but sacrifices healthy bone and ligaments in the lateral and patellofemoral compartments [4]. Given SONK’s typically unicompartmental nature, unicompartmental knee arthroplasty (UKA) emerged as a theoretically attractive bone- and ligament-preserving alternative [2]. However, its suitability for SONK was initially debated due to concerns about lesion size, bone quality, and potential for progression compared to primary OA [3]. 

Early reports on UKA for SONK showed mixed outcomes, with some suggesting higher failure rates compared to TKA, often attributed to suboptimal patient selection or technical errors [5]. However, more recent studies benefiting from improved implant designs, minimally invasive techniques, and stricter selection criteria have reported notably better results [6]. Several mid- to long-term follow-ups have shown good implant survivorship and functional recovery in patients meeting appropriate indications. High patient satisfaction, significant improvement in knee scores, and favorable long-term survival have been consistently documented [1]. Furthermore, the functional advantages of UKA are complemented by potential economic benefits, including lower perioperative costs and faster return to work, making it a cost-effective surgical option. The importance of proper patient selection for achieving these optimal outcomes in SONK cannot be overstated. Contraindications for UKA include inflammatory arthritis, significant patellofemoral disease, and extensive bone loss. Importantly, modern data suggests that when limited to well-selected cases with isolated condylar disease and intact ligamentous structures, UKA can yield outcomes comparable to those seen in primary osteoarthritis [7]. Tibial subsidence and component loosening remain the main causes of late failure, though these are relatively infrequent with current surgical standards [2].

Despite growing evidence supporting UKA in SONK, optimal indications, surgical techniques, and long-term outcomes remain debated. This review synthesizes current evidence to guide surgeons in appropriate patient selection, highlighting both the compelling advantages of UKA in ideal candidates and the specific contraindications that should steer the surgeon towards alternative procedures.

## Review

Epidemiology

SONK primarily affects older adults, with a peak incidence in individuals aged ≥55 years [1]. The condition exhibits a strong female predominance, with reported female-to-male ratios ranging from 2:1 to 3:1 [2,7]. This predisposition is thought to be multifactorial, potentially linked to postmenopausal hormonal changes affecting bone metabolism and biomechanical factors that increase stress on the MFC. While the exact prevalence remains unclear, SONK accounts for a small but clinically significant proportion of knee pain cases in the elderly, with some studies estimating it as the underlying pathology in 0.5-7% of patients undergoing knee arthroplasty [3,5]. Although SONK is an uncommon cause of knee pain in the elderly (approximately 1.5% of cases), it is a clinically significant pathology, responsible for 0.5-7% of knee arthroplasty procedures [1,2].

The MFC is the most commonly affected site, representing >80% of cases, followed by the lateral femoral condyle and, rarely, the tibial plateau [8]. Bilateral involvement is uncommon (<10% of cases), further distinguishing SONK from secondary osteonecrosis (e.g., steroid or alcohol-induced), which often presents with multifocal or bilateral disease [4]. 

Pathogenesis and natural history

The exact cause of SONK remains debated, although several mechanisms are considered contributory. One hypothesis suggests microfractures in the weakened subchondral bone due to osteoporosis or repetitive stress lead to subchondral insufficiency fractures, which impair vascular supply and result in localized bone necrosis [8]. This is supported by MRI findings of bone marrow edema and subchondral fracture lines.

Vascular compromise is another factor, particularly in the MFC, which has a relatively poor blood supply and is prone to ischemia. Histological studies often reveal osteocyte death, marrow necrosis, and trabecular collapse consistent with avascular necrosis [9].

Meniscal dysfunction and altered joint biomechanics also play a role. Degeneration or tears in the medial meniscus can lead to uneven load distribution, increasing stress on the subchondral bone. Varus malalignment further concentrates forces on the medial compartment, predisposing to insufficiency fractures [4].

It is important to distinguish SONK from secondary osteonecrosis, which is linked to systemic conditions such as corticosteroid use, alcohol abuse, or hemoglobinopathies. Unlike SONK, which typically occurs unilaterally in older adults without systemic risk factors, secondary osteonecrosis often presents bilaterally and may extend into the metaphysis [2].

SONK typically follows a progressive course, classified radiographically into four stages (Mont classification) [4]: Stage I: normal X-ray with marrow edema visible on MRI; Stage II: subchondral flattening or cysts; Stage III: crescent sign indicating subchondral collapse; and Stage IV: secondary osteoarthritis with joint space narrowing.

Smaller lesions (<40% of the condyle) may stabilize with conservative treatment. However, larger lesions (>50%) often progress to collapse and secondary arthritis, necessitating surgical intervention [10].

Rationale for UKA over alternatives

SONK poses a significant therapeutic challenge, particularly in selecting an optimal intervention based on disease severity and patient profile. While conservative management, HTO, and TKA remain valid treatment options, UKA offers unique advantages in well-selected patients with localized disease.

In early-stage SONK (Stages I-II), conservative measures such as non steroidal anti inflammatory drugs (NSAIDs), bracing, and activity modification may be attempted. However, outcomes are often suboptimal in patients with larger lesions. Aglietti et al. demonstrated that lesions greater than 5 cm² or involving more than 40% of the MFC were associated with poor nonoperative outcomes, with over 80% progressing to collapse [11]. Similarly, Lotke and Ecker found that patients with Stage III-IV lesions (marked by subchondral collapse or the crescent sign) rarely benefit from conservative approaches and often require surgical intervention [10]. By contrast, UKA provides immediate pain relief and functional improvement, especially in advanced disease stages, helping to avoid the morbidity associated with failed nonoperative management [1].

Historically, HTO has been used to offload the necrotic medial compartment in SONK. However, its utility is limited to early lesions without subchondral collapse. Koshino et al. reported a 94.6% rate of pain relief following HTO, but these results were largely confined to early-stage cases [12]. Hernigou et al. found that HTO failed in 30% of cases with subchondral collapse, likely because it does not directly address the necrotic segment [13]. In comparison, UKA allows for direct resurfacing of the necrotic area, effectively removing the pathological bone [3]. In addition, UKA facilitates earlier weight-bearing and quicker rehabilitation and has shown superior long-term survivorship in late-stage disease, with 10-year survival rates of 89-93% for unicompartmental knee replacement (UKR) versus less than 70% for HTO [2,6].

Although TKA is a definitive solution in patients with bicompartmental or patellofemoral involvement, it may be excessive for isolated medial SONK. Radke et al. observed comparable functional outcomes between TKA and UKA in patients with SONK, but emphasized that UKR better preserved bone stock and native knee kinematics [5]. Furthermore, Bruni et al. reported higher complication rates such as blood loss, infection, and stiffness, with TKA compared to UKA [2]. In terms of patient satisfaction, Heyse et al. found a 97.3% satisfaction rate with UKA versus 90% for TKA, likely due to the minimally invasive nature of UKA and preservation of the cruciate ligaments [1]. The benefits of UKA further include shorter hospital stays, smaller incisions, quicker recovery, and lower morbidity [7].

Despite these advantages, appropriate patient selection is paramount for successful UKA outcomes in SONK. Ideal candidates typically present with isolated MFC involvement, intact ligaments, and a correctable varus deformity of less than 15° [4]. Lesion size should be less than 50% of the condylar width, as larger lesions may necessitate TKA. UKA is contraindicated in cases with metaphyseal extension (which may indicate secondary osteonecrosis), inflammatory arthritis, patellofemoral symptoms, or severe bone loss and ligamentous instability [2].

Surgical techniques and considerations

Preoperative Planning

Imaging assessment is crucial before surgery. MRI is mandatory to confirm the location of the lesion, assess the bone quality, and rule out lateral or patellofemoral compartment involvement [2,4]. Radiographs are used to calculate the condylar ratio, defined as lesion width divided by condylar width on AP views and lesion volume, calculated by multiplying width, height, and depth on MRI [7]. It is essential to exclude metaphyseal extension of the lesion, as this may necessitate a TKA instead of a UKA (2).

Patient selection remains one of the most critical factors influencing UKR outcomes. Ideal candidates should have an intact anterior cruciate ligament (ACL), correctable varus deformity of less than 15°, and full-thickness cartilage in the lateral compartment [14]. Contraindications include inflammatory arthritis, significant patellofemoral joint pain, or ligamentous instability [15].

Surgical Approach

A minimally invasive surgical (MIS) approach is preferred, typically through a medial parapatellar or subvastus incision of 6-8 cm [16]. Patellar eversion is avoided to preserve the extensor mechanism, while quadriceps-sparing methods are employed to reduce postoperative pain and promote faster recovery [17].

During bone preparation, all necrotic bone must be debrided until healthy, bleeding subchondral bone is encountered [3]. Large defects may require cement augmentation [7]. Care must be taken to preserve the posterior femoral cortex to prevent postoperative flexion instability.

Component Positioning

Proper positioning of both tibial and femoral components is essential. For the tibial component, overcorrection should be avoided. The posterior slope should replicate native anatomy, typically between 3° and 7° for medial UKR [18]. Slopes exceeding 10° increase the risk of bearing dislocation or tibial subsidence.

The femoral component must restore the joint line accurately to avoid mid-flexion instability. The flexion-extension alignment should remain within ±5° of neutral, and anterior femoral notching must be avoided to reduce the risk of fracture [14].

Bearing Selection and Fixation

The choice between mobile- and fixed-bearing designs in UKR remains largely a matter of surgeon preference, as long-term studies have shown comparable survivorship and functional outcomes with both [19]. While mobile-bearing systems, such as the Oxford UKR, were designed to reduce polyethylene wear by allowing self-adjusting rotation, they may carry a small risk of bearing dislocation [6]. Fixed-bearing implants, on the other hand, are sometimes preferred for patients with limited range of motion or lower functional demand [1].

With regard to fixation, careful cementation technique is critical for implant longevity. Adequate bone preparation with pulse lavage of cut surfaces and the creation of multiple small drill holes in both femoral and tibial cuts enhances cement penetration into cancellous bone, improving fixation strength. Cement should be applied in a pressurized manner for optimal interdigitation, while care must be taken to avoid excess cement in the posterior recess to prevent soft-tissue impingement [5].

Intraoperative Challenges in SONK

Bone defects are a hallmark of SONK and must be managed based on size. Small defects can be filled with cement, while larger defects, those exceeding 20 cm³, may require bone grafting or metal augmentation [2,7]. Soft tissue balancing is equally important; over-release of the medial collateral ligament (MCL) should be avoided to prevent instability. Intraoperative evaluation of varus-valgus stability throughout the range of motion is essential [10].

Postoperative Protocol

Early mobilization is encouraged. A continuous passive motion (CPM) machine is typically initiated on the first postoperative day. Patients are allowed to bear weight as tolerated with the aid of crutches for two to four weeks [16]. The rehabilitation phase focuses on quadriceps strengthening and recovery of range of motion. Patients are advised to avoid deep squatting for at least 6 weeks to reduce the risk of bearing dislocation [17].

Outcomes of UKA in SONK

Clinical and Functional Outcomes

UKA for SONK yields excellent pain relief and functional improvement in appropriately selected patients. Multiple studies report significant postoperative gains in pain and function scores. Heyse et al. observed a marked improvement in Knee Society Scores (KSS), from a preoperative mean of 85 ± 30 to 173 ± 27 at a mean follow-up of 10.9 years (p < 0.0001) [1]. Bruni et al. reported improvements in WOMAC scores, with postoperative values averaging 12 ± 10.3 and a 90% patient satisfaction rate [2]. Similarly, Ma et al. demonstrated significant Oxford Knee Score (OKS) improvement, from 41.2 ± 7.0 preoperatively to 19.7 ± 4.4 at five years (p < 0.01) [7].

Range of motion (ROM) also shows consistent improvement following UKA. Choy et al. noted correction of flexion contractures from 8.9° to 0.2°, with overall flexion improving from 138.6° to 145.6° [20]. In addition, Pandit et al. reported that more than 90% of patients achieved over 120° of flexion within six weeks postoperatively [16]. Patient satisfaction following UKA is consistently high, Heyse et al. reported a 97.3% satisfaction rate, and Ma et al. found that 91% of SONK patients reported good/excellent results, comparable to osteoarthritis patients [1,7]. Postoperative function also allows for return to moderate activity levels, with 84% of patients able to squat and 91% able to sit cross-legged, as noted by Choy et al. [20].

Implant Survivorship

Mid- to long-term survivorship of UKA in SONK is favorable when strict selection criteria are observed. In appropriately selected patients, Bruni et al. documented a 10-year survivorship rate of 89% (using revision for any reason as the endpoint), while Parratte et al. reported a 96.7% survivorship at 12 years [2,6]. Langdown et al. found 100% implant survival at five years in a cohort of 27 patients [21]. Comparisons with TKA show encouraging results; Radke et al. found comparable functional outcomes between UKA and TKA in SONK patients, with the added benefit of bone stock preservation in UKA [5]. Similarly, Myers et al. demonstrated survival rates of UKA comparable to TKA, particularly when excluding cases of secondary osteonecrosis [22].

Positive prognostic factors influencing survivorship include lesions involving less than 50% of the condylar width, an intact ACL and lateral compartment, and well-aligned implants with 3-7° of tibial slope and minimal varus/valgus deviation [20,23]. Conversely, survivorship may be compromised in cases with large bone defects exceeding 20 cm³, metaphyseal extension (suggesting secondary osteonecrosis), or overcorrection into valgus greater than 5° from native alignment [2].

When comparing fixed- and mobile-bearing designs, most large series and registry data indicate similar long-term survivorship and functional outcomes [24]. While mobile-bearing implants were designed to reduce polyethylene wear, they have been associated with a small but distinct risk of bearing dislocation, occasionally necessitating revision [24]. Fixed-bearing designs avoid this complication and are considered equally durable, with revision rates in modern series comparable to mobile systems. Thus, survivorship differences are less a function of implant design and more dependent on meticulous surgical technique and appropriate patient selection.

Comparative Outcomes: UKA Versus Alternatives

When compared to nonoperative and other surgical options, UKA provides superior outcomes in suitable SONK patients. Aglietti et al. reported an 80% failure rate with conservative treatment in lesions exceeding 5 cm², highlighting the limitations of nonoperative management [11]. UKA, by contrast, enables quicker pain relief and functional recovery. Compared to HTO, UKA demonstrates better reliability and earlier return to function. Koshino et al. reported a 30% failure rate in patients undergoing HTO for advanced SONK (Stages III-IV), whereas UKA provided more predictable outcomes and allowed earlier weight-bearing [4,12].

In head-to-head comparisons with TKA, UKA has shown similar clinical scores. Servien et al. found no significant differences in clinical outcomes between UKA and TKA at five-year follow-up, but noted that UKA patients experienced lower morbidity, including reduced blood loss, infection risk, and faster recovery [18]. UKA also preserves native knee kinematics and bone stock, which can be advantageous for future revisions (Table 1).

**Table 1:** Key Takeaways Table KSS - Knee Society Scores OKS - Oxford Knee Score Percentages (e.g., survivorship, patient satisfaction, complication rates) indicate the proportion of patients with favorable outcomes or specific complications. Arrows (→) indicate improvement from preoperative to postoperative scores (e.g., KSS improved from 85 to 173; OKS improved from 41 to 20, with lower scores reflecting better outcomes).

Outcome Measure	Results	Key Studies
10-year survivorship	89-97%	Bruni et al. [2]
KSS improvement	85 → 173	Heyse et al. [1]
OKS improvement	41 → 20	Ma et al. [7]
Complication rates	Bearing dislocation: 2-9%	Choy et al. [20]
Patient satisfaction	91-97%	Heyse et al. [1]

Complications and failure modes

UKA in SONK is associated with a relatively low complication rate, although some issues warrant attention. Several complications may arise following UKR. Bearing dislocation, reported in 2-9% of cases, is often due to MCL laxity, excessive posterior slope, or improper bearing size. Management includes exchanging for a thicker bearing or, if necessary, revising the procedure [1,2]. Tibial subsidence, which occurs in 4-8% of cases, is linked to osteoporotic bone, excessive slope, or undersizing of the implant. Prevention strategies include achieving adequate cementation and correct alignment [5]. Progression of osteoarthritis, particularly in the lateral compartment, remains a concern but occurs in less than 5% of well-selected cases. Symptomatic progression may eventually require conversion to TKA [6,18].

Careful attention to surgical technique, including avoiding excessive slope and ensuring proper cementation, can mitigate these risks. Patient-reported outcomes strongly reflect the importance of technique, high satisfaction and functional recovery are closely tied to precise implant alignment and appropriate patient selection.

When failure does occur, most revisions can be managed with conversion to TKA, which is typically less complex than revision of a primary TKA, as bone stock and ligament balance are often preserved. Standard primary implants are usually sufficient, although tibial defects may occasionally require grafting or augments. Functional outcomes after conversion are generally comparable to those of primary TKA, although registry data suggest a slightly higher re-revision risk compared with primary arthroplasty [1,5].

Future directions and research gaps

While mid-term outcomes (five to 10 years) of UKA for SONK are encouraging, with 10-year survivorship ranging from 89% to 97%, data beyond 15 years are scarce [2,6]. Longer follow-up is needed to assess late-stage polyethylene wear in mobile-bearing designs, the progression of contralateral compartment osteoarthritis, and comparisons with TKA survivorship in elderly patients. Large-scale multicenter registry studies would help address this limitation. Large-scale multicenter registry studies and prospective trials would help overcome the limitations of small single-center series, providing stronger evidence on implant longevity, complications, and functional outcomes in diverse patient groups.

Patient selection currently relies heavily on MRI findings and lesion size, yet the integration of additional biomarkers could enhance prognostic accuracy. Bone quality assessment using DEXA (dual-energy X-ray absorptiometry) or trabecular bone score may predict subsidence risk, while advanced imaging such as PET-CT (positron emission tomography-computed tomography) or SPECT (single-photon emission computed tomography) could assess metabolic bone activity [25]. Genetic markers may also help identify patients predisposed to spontaneous osteonecrosis [26]. Prospective studies correlating such preoperative parameters with postoperative outcomes are warranted. Future prospective studies correlating these parameters with outcomes could establish more reliable selection algorithms.

Most UKA implants were designed for osteoarthritis rather than osteonecrosis, and design refinements may improve outcomes in SONK. Potential modifications include enhanced fixation surfaces to address compromised subchondral bone, modular augments for larger defects of 20-30 cm³, and alternative bearing materials such as highly crosslinked polyethylene or ceramic [1,7]. Comparative biomechanical studies between standard and augmented UKA designs in necrotic bone could provide valuable guidance. Surgical technique refinements are another area of interest, particularly regarding the extent of bone debridement versus preservation, the use of cement augmentation for large defects, optimal alignment philosophy (kinematic versus mechanical), and the influence of tibial slope on stability and wear [3,18,20].

Management of large lesions remains challenging. Although lesions involving less than 50% of the condyle are generally considered safe for UKA, “borderline” defects of 40-60% may require adjunctive strategies such as bone grafting, custom augments, or patient-specific instrumentation [11,13,23]. Direct comparisons between UKA and TKA for these larger lesions are lacking. In addition, while UKA is associated with reduced hospital stays and fewer early complications compared with TKA, the long-term cost implications of potential revisions remain unclear [14]. Health economic studies, particularly within value-based care frameworks, would help clarify the cost-effectiveness of UKA in SONK [5].

Postoperative rehabilitation protocols also vary, with uncertainty regarding weight-bearing progression after bone grafting, range-of-motion restrictions following cement augmentation, and safe timelines for returning to activity in different age groups [16,27]. Controlled trials comparing accelerated and conservative rehabilitation strategies could standardize care. Finally, the absence of SONK-specific guidelines in major joint replacement recommendations highlights the need for international consensus. This should include clear indications for UKA, standardized outcome measures, and refined classification systems incorporating both MRI and clinical findings [10,21].

In summary, the most pressing research needs include the acquisition of long-term outcome data, refinement of patient selection using advanced diagnostics, optimization of implant design for necrotic bone, standardization of surgical and rehabilitation protocols, cost-effectiveness analyses, and the development of SONK-specific consensus guidelines. Addressing these through coordinated multicenter studies, registry data, and prospective trials will be key to improving UKA outcomes in this complex patient population. Coordinated multicenter registry studies and well-designed prospective trials will be crucial to addressing these gaps and improving outcomes in this complex patient population.

## Conclusions

SONK represents a distinct clinical entity, most often affecting the medial femoral condyle of older adults, with a natural history that frequently progresses to subchondral collapse and secondary osteoarthritis. While conservative management and high tibial osteotomy may provide symptomatic relief in early disease, their effectiveness diminishes once collapse occurs. TKA remains a reliable option for advanced or multicompartmental involvement, but for patients with isolated medial disease, UKA offers a balanced solution, preserving bone stock, restoring native kinematics, and allowing for faster recovery with excellent functional outcomes and survivorship when appropriate selection criteria are applied. Future research should focus on refining patient selection with advanced diagnostics, optimizing implant design for compromised subchondral bone, and standardizing rehabilitation and surgical protocols. The development of SONK-specific guidelines will be essential to unify practice and improve long-term outcomes for this challenging condition.
